# Determination of Carbamate and Organophosphorus Pesticides in Vegetable Samples and the Efficiency of Gamma-Radiation in Their Removal

**DOI:** 10.1155/2014/145159

**Published:** 2014-03-10

**Authors:** Muhammed Alamgir Zaman Chowdhury, Iffat Jahan, Nurul Karim, Mohammad Khorshed Alam, Mohammad Abdur Rahman, Mohammed Moniruzzaman, Siew Hua Gan, Abu Naieum Muhammad Fakhruddin

**Affiliations:** ^1^Agrochemicals and Environmental Research Division, Institute of Food & Radiation Biology, Atomic Energy Research Establishment, Savar, Dhaka 1349, Bangladesh; ^2^Department of Environmental Sciences, Jahangirnagar University, Dhaka 1342, Bangladesh; ^3^Department of Biochemistry and Molecular Biology, Jahangirnagar University, Savar, Dhaka 1342, Bangladesh; ^4^Human Genome Centre, School of Medical Sciences, Universiti Sains Malaysia, 16150 Kubang Kerian, Kelantan, Malaysia

## Abstract

In the present study, the residual pesticide levels were determined in eggplants (*Solanum melongena)* (*n* = 16), purchased from four different markets in Dhaka, Bangladesh. The carbamate and organophosphorus pesticide residual levels were determined by high performance liquid chromatography (HPLC), and the efficiency of gamma radiation on pesticide removal in three different types of vegetables was also studied. Many (50%) of the samples contained pesticides, and three samples had residual levels above the maximum residue levels determined by the World Health Organisation. Three carbamates (carbaryl, carbofuran, and pirimicarb) and six organophosphates (phenthoate, diazinon, parathion, dimethoate, phosphamidon, and pirimiphos-methyl) were detected in eggplant samples; the highest carbofuran level detected was 1.86 mg/kg, while phenthoate was detected at 0.311 mg/kg. Gamma radiation decreased pesticide levels proportionately with increasing radiation doses. Diazinon, chlorpyrifos, and phosphamidon were reduced by 40–48%, 35–43%, and 30–45%, respectively, when a radiation strength of 0.5 kGy was utilized. However, when the radiation dose was increased to 1.0 kGy, the levels of the pesticides were reduced to 85–90%, 80–91%, and 90–95%, respectively. In summary, our study revealed that pesticide residues are present at high amounts in vegetable samples and that gamma radiation at 1.0 kGy can remove 80–95% of some pesticides.

## 1. Introduction

Pesticides such as insecticides, herbicides, fungicides, and acaricides are an abundant and diverse group of chemical compounds. Pesticides are widely applied during cultivation and postharvest storage to improve the quantities and quality of crops and food [1]. The use of pesticides is essential to control pests in horticultural crops and to ensure the production of adequate food supplies for the increasing world population, as well as to control insect-borne diseases. Pesticides are used to decrease crop loss both before and after harvest [2, 3] and to prevent the destruction of edible crops by controlling agricultural pests or unwanted plants, thereby improving food production [4–6]. The increased use of pesticides has led to fears of adverse consequences not only for human health but also for the environment due to pollution.

The general population is exposed to pesticides on a daily basis via dietary ingestion of contaminated food products. Several studies have indicated that certain foods contain higher levels of pesticide residue, such as fruits, juices, and vegetables [7]. Vegetables containing residue concentrations above the prescribed maximum residue level (MRL) may pose a health hazard to unwary consumers [8–11].

Fresh fruits and vegetables are important components of a healthy diet, as they are a significant source of vitamins and minerals. Different types of vegetables are consumed daily by locals in Bangladesh. Among them, eggplant is one of the most common vegetables used in various dishes. Therefore, monitoring pesticide residues in vegetables, particularly in eggplant, may indicate the extent of pesticide contamination that may pose a possible risk to human health.

Several methods can be employed for the removal of various classes of pollutants from contaminated environmental samples [12]. Some of these methods are advanced oxidation processes (AOPs), including UV photolysis, photocatalysis (hydrogen peroxide and ozone), analysis using Fenton's reagent, and radiolysis of water [12–16]. In addition, radiation is one of the most powerful AOPs, in which irradiation with a beam of accelerated electrons or gamma- radiation can decompose various pollutants, such as pesticide residues.

Radiolytic degradation of pollutants has been employed in recent years for treatment of natural waters and wastes of different origins and has also been used for drinking water treatment [17–20]. Moreover, gamma-irradiation is becoming an important technology in the food industry, including food safety concerns such as the preservation of fruits and vegetables to reduce pathogenic microbes [21]. On the other hand, even though radiation of food has been investigated by many scientists, limited studies have focused on the effect of gamma-radiation for the removal of pesticide residues [22–24].

In recent years, carbamate and organophosphorus pesticides have become increasingly important due to their broad spectrum of activity, their relatively low persistence, and their generally low mammalian toxicity when compared to organochlorine pesticides [25–27]. Although carbamate and organophosphorus pesticides are extensively used by Bangladeshi farmers during the cultivation of crops and vegetables, there is very little information on the incidence of vegetable samples that have been contaminated with these pesticides and whether irradiation of foods, specifically vegetable samples, prior to their consumption is an efficient method for the removal of such contaminants.

Thus, the aim of the present study was to determine the residual levels of carbamate and organophosphorus pesticides in samples of a vegetable widely consumed in Bangladesh, namely, eggplant. The effect of radiation treatment on the removal of pesticide residues from four types of vegetables that are commonly consumed raw, namely, capsicum, cucumber, carrot, and tomato, was also investigated.

## 2. Materials and Methods

### 2.1. Chemicals and Reagents

The carbaryl (98.5%), carbofuran (99.5%), diazinon (99.0%), dimethoate (97.5%), parathion (98.5%), phenthoate (98.5%), phosphamidon (99.0%), pirimicarb (97.5%), and pirimiphos-methyl (97.5%) standards (Table 1) used in this study were of reference grade and were purchased from Dr. Ehrenstorfer GmbH, 86199 Augsburg, Germany. The solvents, such as acetone (BDH, England), n-hexane (Merck, Germany), and diethyl ether (BDH, England), were of analytical grade, while the acetonitrile (ACN) (Scharlau, EU) was of high performance liquid chromatography (HPLC) grade.

### 2.2. Collection and Preservation of Samples

To monitor the pesticides present in vegetable samples, eggplant (*Solanum melongena*) samples (*n* = 16) were collected from four different markets in the Gulshan-2 area, Dhaka, Bangladesh. The vegetable samples that were used to investigate the effects of radiation treatment on the removal of pesticides were capsicum (*Capsicum annum*), cucumber (*Cucumis sativus*), and tomato (*Solanum lycopersicum*). These vegetables were selected because they are usually eaten raw.

Only fresh, high-quality vegetables that were free from blemishes or rot were used. Following collection, the samples were refrigerated at 4 ± 1°C overnight and analyzed the next day. To reduce variability, all of the vegetable samples used in the study were collected within similar areas.

### 2.3. Sample Extraction for Pesticide Analysis

Sample preparation was conducted by following the methods described by [28, 29]. An amount of sample (200 g) was chopped, and a small amount (20 g) was then macerated with 50 mL of ethyl acetate, hexane, and acetone (3 : 1 : 1). Anhydrous sodium sulfate (20 g) was added to remove water before the addition of 0.05–0.10 g AAC for the removal of soluble plant pigments. The mixture was further macerated at full speed for 3 min using an Ultra-Turrax macerator (IKA-Labortechnik, Janke & Kunkel GmBH & Co., KG, Germany). The samples were then centrifuged for 5 min at 3000 rpm, and the supernatant was transferred to a clean graduated cylinder for volume measurement. The organic extract was concentrated to 5 mL using a vacuum rotary evaporator (Rotavapor-R 215, Buchi, Switzerland) at 250 mbar with water bath at 45°C. The extraction process was followed by a cleanup step using column chromatography with Florisil (60–100 mesh, Sigma, USA, analytical grade) to remove any residual components that may interfere with the HPLC detector system.

### 2.4. Sample Preparation for Radiation Treatment

The vegetable samples were carefully washed with running tap water, as usually practiced in domestic kitchens. After washing, the stems of all samples were removed. Cucumbers were first peeled with a peeler, followed by uniform slicing using a sterile knife on a clean chopping board. The tomatoes were sliced without being peeled, while capsicum was directly chopped.

### 2.5. Radiation Treatment

The samples were packed into sterilized (15 kGy radiation dose) low-density polyethylene (LDPE) plastic bags before being sealed with a sealer. Two samples were prepared for each type of vegetables. The packets were individually labeled, and two different radiation doses (0.5 and 1.0 kGy) were applied to each. In this study, a 1850 terabecquerel (50 kCi) ^60^Co gamma-irradiator was used as the radiation source. Nonirradiated sample of each type of vegetables was kept as control sample. After the radiation treatment, both the irradiated and the nonirradiated samples were analyzed for the presence of pesticides following the above method.

### 2.6. Cleaning of Extracts

The samples were cleaned following the method described by [30]. Briefly, the cleaning of acetone extract was performed using Florisil column chromatography. The Florisil (60–100 mesh) was activated at 200°C for 6 h and was subsequently deactivated with 2% distilled water. The top 1.5 cm of the 0.6 cm diameter Florisil column was packed with anhydrous sodium sulfate. Elution was performed with a solvent mixture of double distilled hexane (65%) and dichloromethane (35%) at 5 mL/min. The eluent was concentrated to a small volume (1-2 mL) using a rotary vacuum evaporator and transferred to a vial. Any residual solvent was completely removed under a gentle flow of nitrogen. The evaporated sample was reconstituted to a total volume of 1 mL by dissolution in acetonitrile prior to HPLC injection. The procedure was similarly conducted for all vegetable samples.

### 2.7. HPLC Analysis

Following the cleaning of extract, aliquots of the final solution were quantified using a (Shimadzu) LC-10 ADvp HPLC, equipped with an SPD-M 10 Avp attached to a photodiode array detector (Shimadzu SPD-M 10 Avp, Japan) (200–800 nm). The analytical column was a C18 Reverse Phase from Alltech (250 × 4.6 mm, 5 *μ*m) that was maintained at 30°C in a column oven. A combination of 70% ACN and 30% water was used as the mobile phase, running with a flow rate of 1.0 mL/min. All solvents were of HPLC grade and were filtered using a cellulose filter (0.45 *μ*m) prior to use.

**Figure 1:**
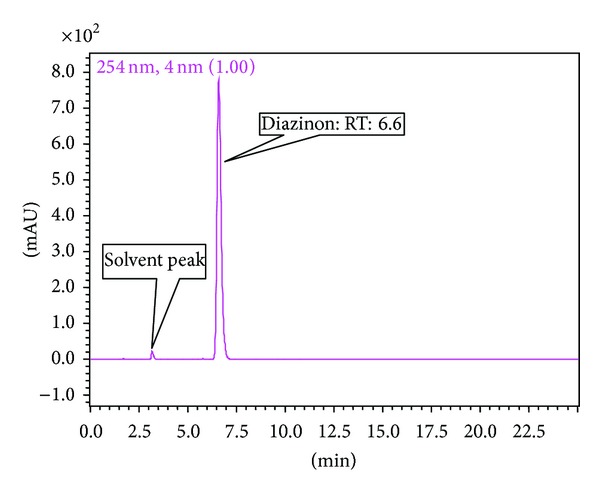
Typical chromatogram of a diazinon standard injected at 100 *μ*g/mL (RT = retention time 6.6 min).

Prior to HPLC analysis, the samples were passed through a 0.45 *μ*m nylon syringe filter (Alltech Assoc) before being manually injected (20 *μ*L) each time. Suspected pesticides were identified based on the retention times of the pure analytical standards. Quantification was performed based on the method described by [28] (Figure 1).

### 2.8. Calibration Curve

The calibration curves for carbofuran, carbaryl, pirimicarb, phenthoate, diazinon, parathion, dimethoate, phosphamidon, and pirimiphos-methyl were determined using five different concentrations (5, 10, 20, 40, and 100 *μ*g/mL) in duplicate.

### 2.9. Quality Control and Quality Assurance

Quality control and quality assurance were incorporated into the analysis. The accuracy and precision were validated in accordance with the European Commission (EC) guidelines [31]. The precision was expressed as the relative standard deviation (RSD). Accuracy can be measured by analyzing samples with known concentrations and comparing the measured values with the actual spiked values. For the recovery experiments, pesticide-free samples (20 g) were spiked in triplicate (*n* = 3), after homogenization by the addition of appropriate volumes of pesticides standards at two different levels (0.05 and 0.50 *μ*g/mL). Control samples were processed along with spiked ones. The mixture was left standing for 1 h to allow equilibration. The processes of extraction and cleanup of pesticide residues were similar as described above [28, 30]. The mean percentage recoveries ranged from 86% to 99% while precision ranged from 4.45% to 14.54%.

Percentage recovery = [CE/CM ×100], where CE is the experimental concentration determined from the calibration curve and CM is the spiked concentration.

### 2.10. The Limit of Quantification (LOQ) and Limit of Detection (LOD)

LOQ was defined as the lowest concentration of the analyte that could be quantified with acceptable precision and accuracy. The LOD was defined as the lowest concentration of the analyte in a sample that could be detected but not necessarily quantified. The LOQ and LOD were evaluated as signal-to-noise ratios (S/N) of 10 : 1 and 3 : 1, respectively, and were obtained by analyzing unspiked samples (*n* = 10) [32]. In the present study, the LOD and LOQ were 0.001 mg/kg and 0.003 mg/kg, respectively.

## 3. Results and Discussion

### 3.1. Analysis of Carbamate and Organophosphorus Residues

**Figure 2:**
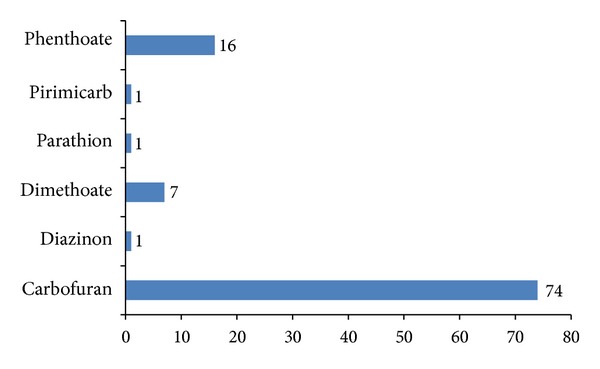
Types of pesticides and the frequency of appearance of given pesticides in the samples (%).

This is the first study to determine the occurrence of organophosphorus and carbamate residues in eggplant samples (*Solanum melongena*) collected from four different markets in the Gulshan-2 area in Dhaka. Pesticide residues were detected in 50% of the 16 samples, and approximately 19% of the total samples exceeded the MRL level provided by the World Health Organisation (WHO) or Food and Agricultural Organisation (FAO). Two samples (VS-15 and VS-16) were contaminated with carbaryl and pirimicarb, while another sample (VS-14) contained carbofuran. In addition to the detected carbamates, six organophosphorus pesticides (diazinon, dimethoate, parathion, phenthoate, phosphamidon, and pirimiphos-methyl) were also detected in seven of the eggplant samples, with some exceeding the MRL level set by FAO/WHO. Among the pesticides detected, carbofuran was present in most (74%) of the samples while phenthoate and dimethoate were present in 16% and 7% of the samples, respectively (Figure 2). The concentrations of the remaining three pesticides were within safe limits.

Due to the long persistence nature of organochlorine pesticides, these have recently been eliminated from agricultural practices in many countries, including Bangladesh [33]. However, the use of carbamates and organophosphorus pesticides has increased because their low persistence has led to claims that they are less harmful to the environment. Therefore, in the present investigation, we focused on carbamates and organophosphates in vegetables normally eaten raw because the exposure of the consumer to the pesticides will be greater for vegetables which are eaten raw than cooked one.

**Figure 3:**
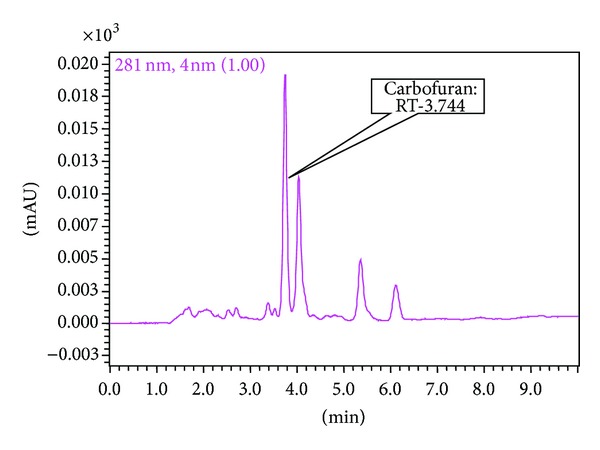
Chromatogram of VS-14 showing the presence of carbofuran (RT: 3.74 min).

Among the carbamate pesticides, carbofuran was detected at a very high concentration (1.86 mg/kg) in a single sample (VS-14) (Figure 3). Contrary to the findings from [34], who did not detect any carbofuran or carbaryl residues in eggplant samples, carbaryl was detected in two eggplant samples (VS-15 and VS-16) at 0.003 and 0.006 mg/kg, respectively (Table 2). This variation may be due to the use of different eggplant sources to supply the market. Other than carbaryl, the two similar samples (VS-15 and VS-16) were also contaminated with pirimicarb at 0.008 and 0.007 mg/kg, respectively. In some cases, the detected pesticide residue concentrations exceeded the recommended limit set by WHO; this can be dangerous to the health of consumers.

**Figure 4:**
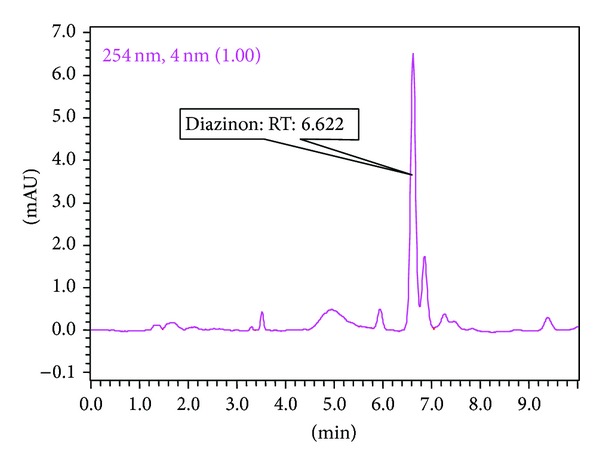
Chromatogram of VS-3 showing the presence of diazinon (retention time 6.6 min).

Six different organophosphorus pesticide residues were analyzed to determine their levels in the collected eggplant samples. Among all the organophosphorus pesticide residues analyzed, the highest phenthoate concentration observed was in sample VS-14 (0.311 mg/kg), followed by sample VS-16 (0.077 mg/kg) (Tables 2 and 3). The sample VS-3 contained two pesticides (diazinon and parathion) at 0.022 (Figure 4) and 0.006 mg/kg. The level of the detected parathion was lower than that detected in some eggplant samples collected from Dhaka, Bangladesh (0.32 mg/kg), in a previous study [34]. Dimethoate was detected in a single sample (VS-8) at 0.183 mg/kg, while phosphamidon and pirimiphos-methyl were detected in samples VS-6 and VS-3 at 0.022 and 0.008 mg/kg (Tables 2 and 3), respectively. Phenthoate and phosphamidon were present at levels higher than the values recommended by the FAO/WHO. In comparison to our result, eggplant samples from India had a lower dimethoate level (0.030 mg/kg) but contained a higher phosphamidon level (0.038 mg/kg), as reported by [35].

### 3.2. Removal of Pesticide Residues in Vegetables Using Gamma-Radiation

The persistence of pesticide residues is a complex matter affected not only by the chemical and physical characteristics of the parent compound and its degradation products but also by the nature of the formulation applied, the adsorbents, and the type of solvents employed. Some types of plants have waxy surfaces that tend to trap sprayed pesticides, thereby making pesticides more resistant to removal, and they would be as true surface residues. Although washing, peeling, and cooking remove a large amount of pesticides during food processing, some studies have indicated that they are inefficient in reducing pesticide residues below the MRL value. For example, [36] reported that quinalphos residues in cauliflower were reduced only to some extent by various home processing methods such as washing and cooking. It has been suggested that the inefficiency of home processes for decontaminating treated cabbage may be due to the strong adsorption properties of quinalphos and chlorpyrifos [37].

Due to the persistent nature of some pesticides and the inefficiency of conventional methods of pesticide removal, additional steps to remove pesticides and their degradation products should ideally be incorporated. Unfortunately, even though the additional steps are important, they are not normally employed because they do not enhance food value. In addition, few studies have investigated the effectiveness of additional steps in removing pesticides, including the use of gamma-radiation.

In the present study, three vegetables that are normally eaten raw in Bangladesh were selected to determine the best radiation dose useful for the reduction of pesticide residues to safer levels. We have selected the WHO recommended doses of radiation for processed and peeled vegetables at 0.5–1.0 kGy, as opposed to the higher levels recommended for unprocessed vegetables at 2.5 kGy [38].

**Figure 5:**
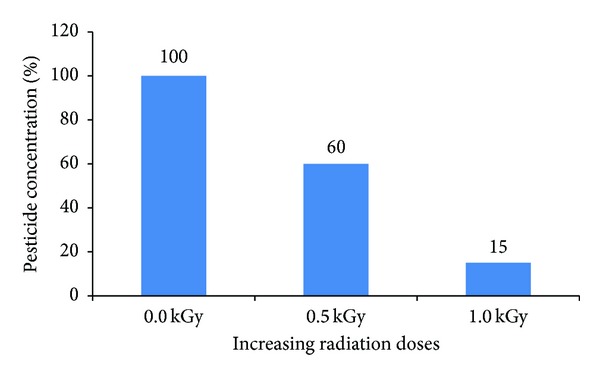
Reduction of diazinon on capsicum with increasing radiation doses.

In this study, the collected samples contained three different pesticides which are diazinon in capsicum, chlorpyrifos in cucumber, and phosphamidon in tomato (Table 4). When the samples were treated with 0.5 kGy gamma-radiation, there was a reduction in the total amount of pesticides present; the degree of reduction varies with the pesticide type. For example, chlorpyrifos, diazinon, and phosphamidon were reduced by 35–43%, 40–48%, and 30–45%, respectively, when radiation strength of 0.5 kGy was utilized (Figure 5). However, when the radiation dose was increased to 1.0 kGy, the levels were reduced to 80–91%, 85–90%, and 90–95%, respectively. The ideal radiation dose is probably 1.0 kGy because when radiation was applied at 0.5 kGy, the highest reduction rate was only 40–48% for diazinon, but at a 1.0 kGy radiation dose, the highest reduction rate could reach up to 90–95% for phosphamidon (Table 4). Furthermore, based on the International Atomic Energy Agency (IAEA) criteria, irradiation doses of up to 1.5 or 2.0 kGy doses are deemed to be safe as they do not affect the quality and appearance of fresh vegetables [39].

Continuous monitoring of residual pesticide levels in different environmental samples and the study of the best method for their removal is important to understand the level of contamination and to determine remedial actions.

## 4. Conclusion

This study reveals the presence of carbamate and organophosphorus residues in eggplant samples collected from four different markets in the Gulshan-2 area in Dhaka; some of these residues exceeded the MRL limits. Our results also indicated that pesticide levels decreased with increasing radiation doses and varied with pesticide type. Continuous monitoring of residual pesticide levels in different vegetable samples is important for their safe consumption.

## Figures and Tables

**Table 1:** Structures, chemical properties, functions, DT_50_, and LD_50 _of the pesticides investigated in this study.

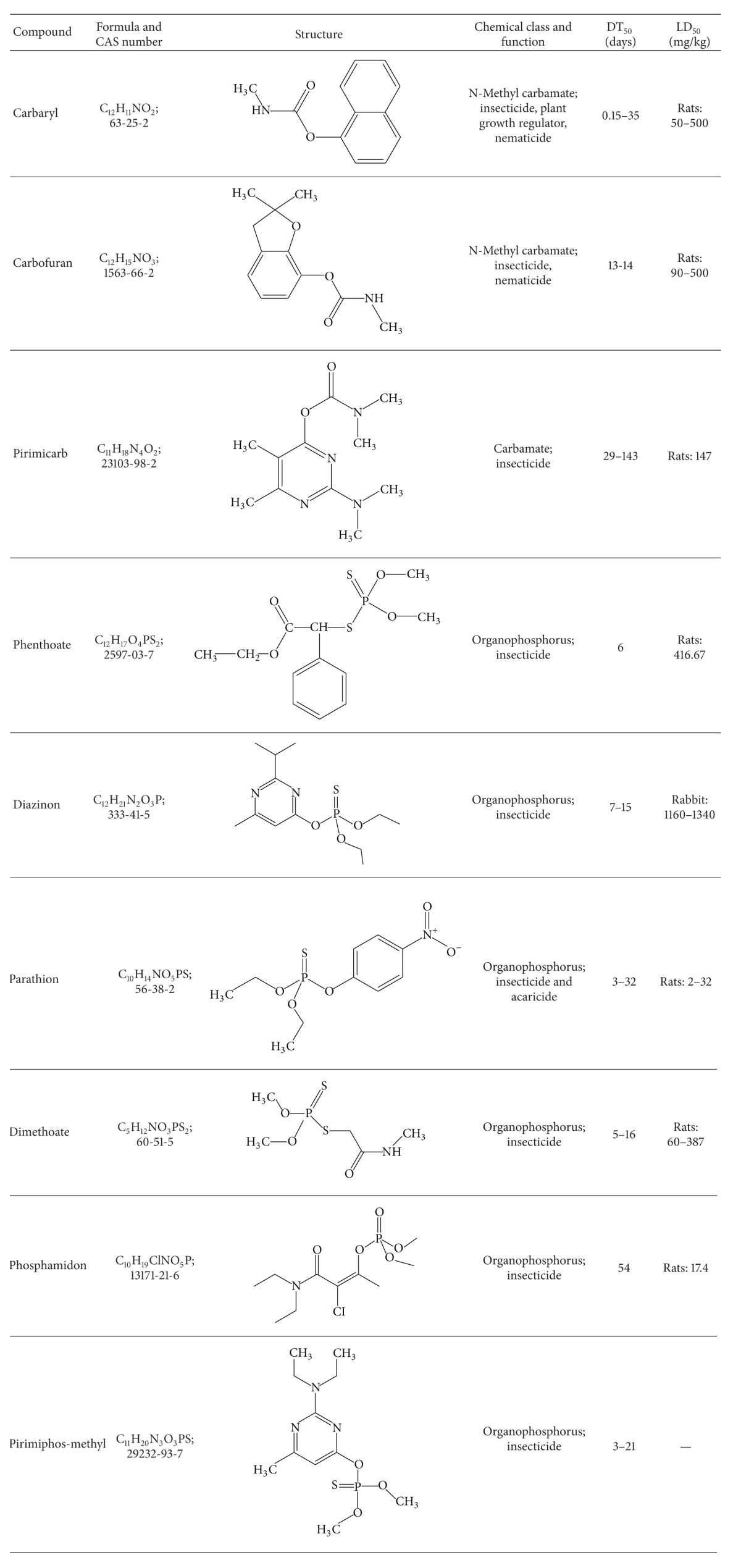

DT_50_: degradation time for 50% of a compound; LD_50_: lethal dose for 50% of the animal population.

**Table 2:** Organophosphorus and carbamate pesticide residues present in vegetable samples.

Sample ID	Phenthoate (mg/kg)	Diazinon (mg/kg)	Parathion (mg/kg)	Dimethoate (mg/kg)	Phosphamidon (mg/kg)	Pirimiphos-methyl (mg/kg)	Carbaryl (mg/kg)	Carbofuran (mg/kg)	Pirimicarb (mg/kg)
VS-3	BDL	0.022	0.006	BDL	BDL	0.008	BDL	BDL	BDL
VS-6	BDL	BDL	BDL	BDL	0.022	BDL	BDL	BDL	BDL
VS-7	BDL	BDL	BDL	BDL	BDL	BDL	BDL	BDL	BDL
VS-8	BDL	BDL	BDL	0.183	BDL	BDL	BDL	BDL	BDL
VS-14	0.311	BDL	BDL	BDL	BDL	BDL	BDL	1.86	BDL
VS-15	BDL	BDL	BDL	BDL	BDL	BDL	0.003	BDL	0.008
VS-16	0.077	BDL	BDL	BDL	BDL	BDL	0.006	BDL	0.007

Table shows only samples that were positive for organophosphate residues. VS: vegetable sample; BDL: below detection limit; limit of detection (LOD): 0.001 mg/kg; limit of quantification (LOQ): 0.003 mg/kg.

**Table 3:** MRL levels and samples with pesticide levels above MRLs.

Sample ID	Carbofuran	Phenthoate	Phosphamidon
VS-6	—	—	0.022
VS-14	1.860	0.311	—
VS-16	—	0.077	—
MRL	0.020	0.010	0.010

Limit of detection (LOD): 0.001 mg/kg; limit of quantification (LOQ): 0.003 mg/kg.

**Table 4:** Percentage of pesticides removed when radiation is applied at two different doses.

	Vegetable sample	Pesticides	Percentage reduced at 0.5 kGy (%)	Percentage reduced at 1.0 kGy (%)
1	Capsicum	Diazinon	40–48	85–90
2	Cucumber	Chlorpyrifos	35–43	80–91
3	Tomato	Phosphamidon	30–45	90–95
